# Evaluating Pro- and Anti-inflammatory Biomarkers for Predicting Type 2 Diabetes Mellitus in the Geriatric Population and Correlation With Clinical and Biochemical Parameters

**DOI:** 10.7759/cureus.77896

**Published:** 2025-01-23

**Authors:** Sartaj Hussain, Suraj Singh Yadav, K K Sawlani, Kauser Usman, Sanjay Khattri

**Affiliations:** 1 Pharmacology, All India Institute of Medical Science, Jammu, Vijaypur, IND; 2 Biotechnology, National Forensic Sciences University, Gandhinagar, IND; 3 Internal Medicine, King George's Medical University, Lucknow, IND; 4 Pharmacology, King George's Medical University, Lucknow, IND

**Keywords:** crp, elderly, il-6, tnf-α, type 2 diabetes mellitus

## Abstract

Background

Type 2 diabetes mellitus (T2DM) and aging are associated with inflammation. Our study investigates the diagnostic accuracy of pro- and anti-inflammatory biomarkers in predicting T2DM in the geriatric population and the relation of these biomarkers with clinical and biochemical parameters.

Methods

This case-control study included age-gender matched, 100 treatment-naïve T2DM cases, and 100 healthy controls. Clinical, biochemical, and anthropometric parameters were comprehensively profiled. The biomarkers were quantified by enzyme-linked immunosorbent assay (ELISA) methods.

Results

In the T2DM group, serum analyses revealed markedly elevated levels of C-reactive protein (CRP), tumor necrosis factor-alpha (TNF-α), interleukin-6 (IL-6), and cortisol, along with notably reduced concentrations of interleukin-10 (IL-10) and adiponectin compared to controls. Despite lower mean insulin-like growth factor-1 (IGF-1) levels in the T2DM group, the differences did not reach statistical significance. The risk of T2DM increased with elevated levels of CRP (odds ratio (OR): 2.69), TNF-α (OR: 1.20), IL-6 (OR: 1.83), and cortisol (OR: 1.01), while higher concentrations of adiponectin (OR: 0.81) and IL-10 (OR: 0.88) were associated with reduced risk (P < 0.05). CRP, TNF-α, IL-6, cortisol, adiponectin, and IL-10 all showed significant area under the curve (AUC) values (P < 0.05), confirming their diagnostic potential. Conversely, insulin-like growth factor-1 (IGF-1) did not reveal any significant diagnostic utility (P=0.327). The optimal cutoff values to diagnose T2DM in the geriatric population determined using the Youden index were as follows: CRP (>3.80 μg/ml), TNF-α (>22.90 pg/ml), IL-6 (>4.67 pg/ml), cortisol (>103.15 ng/ml), adiponectin (≤7.42 μg/ml), and IL-10 (≤5.19 pg/ml).

Conclusion

This study reveals that pro- and anti-inflammatory biomarkers can predict T2DM, have diagnostic potential and, serve as guidance for future therapeutic strategies.

## Introduction

Type 2 diabetes mellitus (T2DM) is a public health challenge worldwide. Insulin resistance plays a central role in the pathogenesis of T2DM, which leads to chronic low-grade inflammation and impairs glucose metabolism [1]. Inflammaging, age-related, chronic low-grade, sterile inflammation, and T2DM are associated with inflammation and imbalance between pro- and anti-inflammatory biomarkers via the nuclear factor-κB (NF-κB) pathway. T2DM is considered an accelerated model of aging, and biomarkers related to inflammaging can accelerate disease progression [2,3].

Pro-inflammatory markers like C-reactive protein (CRP), tumor necrosis factor-alpha (TNF-α), interleukin-6 (IL-6), as well as anti-inflammatory markers, such as adiponectin and interleukin-10 (IL-10), play a significant role in the inflammatory processes of inflammaging and T2DM [4,5]. Pro-inflammatory markers promote insulin resistance and beta-cell dysfunction, while anti-inflammatory markers improve insulin sensitivity and metabolic balance [5-7]. Cortisol plays a central role in stress response and metabolic regulation, and increased cortisol levels are linked to insulin resistance, which further aggravates diabetes in the geriatric population [8]. Furthermore, insulin-like growth factor-1 (IGF-1), associated with both aging and metabolic regulation, plays a central role in the pathogenesis of T2DM by affecting glucose metabolism and cellular aging [9].

The aim of this study is to evaluate the diagnostic accuracy of pro- and anti-inflammatory biomarkers in predicting T2DM in the geriatric population and their correlation with clinical and biochemical parameters. This study investigates the predictive power of CRP, TNF-α, IL-6, cortisol, adiponectin, and IL-10 in diagnosing T2DM in the geriatric North Indian population. The diagnostic abilities and clinical and biochemical relationship of these biomarkers may help improve diagnostic strategies and potential targets of new treatments.

## Materials and methods

Study design, selection of subjects, and ethical aspects

This prospective age-gender-matched, case-control study was carried out at a tertiary care teaching hospital in North India. The study enrolled 200 participants from Northern India, comprising 100 healthy controls and 100 individuals diagnosed with type 2 diabetes mellitus (T2DM) without the presence of any clinical cause of inflammation, all recruited from the outpatient department of internal medicine and health awareness camps. The healthy controls in this study were individuals aged 60 years and older, of either sex, without diabetes, not on regular medication for any disease, and free from chronic physical, congenital, metabolic, cardiac, respiratory, inflammatory, or psychological illnesses, with fasting plasma glucose (FPG) < 100 mg/dL and glycated hemoglobin (HbA1c) < 6.5%. This study screened treatment naïve type 2 diabetes mellitus (T2DM) patients for eligibility, adhering to the 2017 International Diabetes Federation guidelines [10]. Inclusion criteria encompassed age ≥60 years and a confirmed T2DM diagnosis, while exclusion criteria comprised T1DM, hepatic, respiratory, autoimmune, and inflammatory diseases, genetic disorders, cancer, insulin therapy, and bacterial/viral infections. Approval for the protocol (Ref. code: 92nd ECM II A/P12) was obtained from the Institutional Ethics Committee. Written informed consent was acquired from each subject, and the execution of all procedures strictly adhered to the ethical principles set forth in the Declaration of Helsinki.

Data collection, clinical and biochemical measurements

After overnight fasting, 5 mL of venous blood was obtained from antecubital veins through venipuncture. The blood sample was divided into three portions: 2 mL into ethylenediaminetetraacetic acid (EDTA) vial for the estimation of glycated hemoglobin (HbA1c), 1 mL into a fluoride vial for the assessment of fasting plasma glucose (FPG), and the remaining 2 mL into a plain vial to facilitate serum separation for lipid profile and enzyme-linked immunosorbent assay (ELISA). Lipid profile, FPG, and HbA1c were measured on the day of sample collection. The remaining serum fraction, stored at -20°C, was designated for ELISA testing. Fasting plasma glucose (FPG), total cholesterol (TC), triglycerides (TG), and high-density lipoprotein cholesterol (HDL-C) levels in serum samples were estimated by auto-analyzer (Selectra pro-XL Clinical Chemistry System, The Netherlands) and relevant kits. The quantification of HbA1c was achieved through Bio-Rad D-10™ (Bio-Rad Laboratories, Inc., Hercules, California) high-performance liquid chromatography (HPLC) and its associated kit. Low-density lipoprotein cholesterol (LDL-C) levels were determined by applying the Friedewald equation. The serum concentrations of insulin, C-reactive protein (CRP), tumor necrosis factor-alpha (TNF-α), interleukin-6 (IL-6), cortisol, insulin-like growth factor-1 (IGF-1), interleukin-10 (IL-10), and adiponectin were assessed using sandwich enzyme-linked immunosorbent assay (ELISA) with commercially available kits. The kits employed were sourced from insulin, CRP, IL-6, IL-10, IGF-1, and adiponectin kits obtained from RayBiotech, Peachtree Corners, USA; TNF-α from Invitrogen, Waltham, USA; and cortisol from Abcam, Waltham, USA. The ELISA tests were performed in accordance with the manufacturer's protocols. Homeostatic model assessment of insulin resistance (HOMA-IR) and homeostatic model assessment of beta-cell function (HOMA-B) were computed based on the values of fasting insulin and FPG, utilizing the standard formula.

Statistical analysis

The results are presented as mean±standard deviation for continuous variables and as number (%) for categorical variables. Statistical comparisons between continuous and categorical variables were conducted using unpaired Student’s t-test and the χ^2^ test, respectively. The association of pro- and anti-inflammatory markers with other variables was investigated through univariate linear regression analysis. For variables with a P-value ≤ 0.10 in the univariate regression analysis, multivariate linear regression using the forward selection method was applied to identify the independent biomarker-level predictors. An odds ratio (OR) with a 95% confidence interval (CI) was used to express the risk. Binary logistic regression analysis was applied to calculate the odds ratio (OR) for serum pro- and anti-inflammatory markers. To determine the diagnostic accuracy of pro- and anti-inflammatory markers, we performed receiver operating characteristic (ROC) curve analysis, including the estimation of the area under the curve (AUC). The Youden index was used to determine the optimal cut-off value of biomarkers to determine their diagnostic accuracy. All statistical analyses except ROC curve analysis were conducted using SPSS version 25.0 software (SPSS Inc., Chicago, IL, USA). The ROC curve analysis was performed using MedCalc Statistical Software version 20.013 (MedCalc Software, Ostend, Belgium). Two-tailed p-value < 0.05 was considered significant.

## Results

The study included 100 patients with treatment naïve type 2 diabetes mellitus (T2DM) and 100 healthy control subjects. The demographic, clinical, and biochemical parameters of the case and control groups are shown in Table 1. The mean age and gender proportion were comparable between both groups. Body mass index (BMI), systolic blood pressure (SBP), diastolic blood pressure (DBP), fasting plasma glucose (FPG), HbA1c, total cholesterol (TC), triglycerides (TG), low-density lipoprotein cholesterol (LDL-C), fasting insulin, and homeostatic model assessment of insulin resistance (HOMA-IR) were elevated in T2DM group as compared to the control group. Moreover, the T2DM group demonstrated significantly increased serum levels of C-reactive protein (CRP), tumor necrosis factor-alpha (TNF-α), interleukin-6 (IL-6), and cortisol, together with significantly reduced levels of interleukin-10 (IL-10) and adiponectin in comparison to the control group. Serum insulin-like growth factor-1 (IGF-1) level was lower in the T2DM group than in the control group, but this difference was statistically insignificant.

**Table 1:** Demographic, clinical, and biochemical parameters of type 2 diabetes mellitus and control subjects. The results are represented as mean±SD or percentage. The unpaired Student’s t-test or chi-square test was used for comparing data. BMI: body mass index; CRP: C-reactive protein; DBP: diastolic blood pressure; FPG: fasting plasma glucose; HbA1c: glycated hemoglobin; HDL-C: high-density lipoprotein cholesterol; HOMA-IR: homeostasis model assessment of insulin resistance; HOMA-B: homeostasis model assessment of b-cell function; IGF-1: insulin-like growth factor 1; IL-6: interleukin-6; IL-10: interleukin-10; LDL-C: low-density lipoprotein cholesterol; SBP: systolic blood pressure; SD: standard deviation; TC: total cholesterol; TNF-α: tumor necrosis factor-alpha.

Parameters	Control (n=100)	Case (n=100)	P-value
Age (Years)	70.2±5.21	70.82±6.17	0.444
Gender (Male/Female)	55/45	58/42	0.669
Smoking (Yes/No)	19/81	28/72	0.133
Drinking (Yes/No)	12/88	19/81	0.171
Diet (Veg/Non-Veg)	46/54	49/51	0.671
Age at diagnosis (years)	-	55.93±6.87	-
Duration of DM (years)	-	14.89±7.65	-
BMI (kg/m^2^)	23.9±3.41	26.56±3.97	<0.001
Systolic BP (mmHg)	130.32±14.8	143.64±23.55	<0.001
Diastolic BP (mmHg)	82.26±7.76	87.86±13.37	<0.001
FPG (mg/dl)	94.52±5.97	177.53±40.65	<0.001
HbA1c (%)	5.28±0.58	8.27±1.75	<0.001
Total cholesterol (mg/dl)	185.84±29.97	194.06±39.92	0.101
Triglyceride (mg/dl)	140.26±60.89	183.44±66.1	<0.001
HDL-C (mg/dl)	53.55±9.2	44.43±11.41	<0.001
LDL-C (mg/dl)	104.23±28.36	112.94±40.45	0.080
Fasting insulin (μIU/mL)	14.1±9.79	25.64±12.21	<0.001
HOMA-IR	3.31±2.35	11.56±7.27	<0.001
HOMA-B (%)	165.99±111.98	85.79±43.27	<0.001
CRP (μg/ml)	2.48±1.05	7.18±3.66	<0.001
TNF-α (pg/ml)	16.08±6.63	30.36±11.87	<0.001
IL-6 (pg/ml)	3.80±1.85	9.74±5.68	<0.001
Cortisol (ng/ml)	102.09±62.89	152.09±64.37	<0.001
Adiponectin (mg/mL)	9.85±4.97	6.14±3.65	<0.001
IL-10 (pg/mL)	7.44±4.10	5.73±3.21	0.001
IGF-1 (ng/ml)	173.54±63.71	167.21±69.24	0.501

Univariate linear regression analysis was applied to investigate the associations between serum biomarker levels and various parameters. The results of univariate linear regression are presented in Table 2. CRP showed a significant positive correlation with BMI, SBP, DBP, FPG, HbA1c, TC, TG, fasting insulin, and HOMA-IR, while it was inversely correlated with HDL-C and HOMA-B (P < 0.05). TNF-α and IL-6 showed a significant positive association with age, BMI, SBP, DBP, FPG, HbA1c, TG, fasting insulin, and HOMA-IR, along with an inverse correlation with HDL-C and HOMA-B (P < 0.05). Adiponectin displayed statistically relevant negative associations with BMI, SBP, DBP, FPG, HbA1c, TG, fasting insulin levels, and HOMA-IR, concurrently showing a significant positive correlation with HDL-C (P < 0.05). IL-10 displayed significant inverse correlations with FPG, HbA1c, TC, TG, LDL-C, fasting insulin, and HOMA-IR, whereas IGF-1 demonstrated a negative relation with DBP and HOMA-IR (P < 0.05).

**Table 2:** Univariate linear regression analysis of pro- and anti-inflammatory markers. Data was calculated by univariate linear regression analysis and represented as β (beta coefficient), standardized regression coefficients, and P-value. DM: diabetes mellitus; BMI: body mass index; CRP: C-reactive protein; FPG: fasting plasma glucose; HbA1c: glycated hemoglobin; HDL-C: high-density lipoprotein cholesterol; LDL-C: low-density lipoprotein cholesterol; HOMA-IR: homeostasis model assessment of insulin resistance; HOMA-B: homeostasis model assessment of b-cell function; IL-6: interleukin-6; IL-10: interleukin-10; TNF-α: tumor necrosis factor-alpha.

	CRP		TNF-α		IL-6		Cortisol		Adiponectin		IL-10		IGF-1	
Parameters	β	P	β	P	β	P	β	P	β	P	β	P	β	P
Age	0.13	0.067	0.16	0.025	0.20	0.004	0.02	0.741	-0.04	0.544	-0.07	0.313	-0.03	0.663
Age at diagnosis	0.07	0.462	0.08	0.406	0.14	0.156	-0.10	0.309	-0.13	0.199	-0.15	0.144	0.08	0.432
Duration of DM	0.11	0.297	0.09	0.360	0.12	0.228	0.12	0.235	0.07	0.509	0.17	0.086	-0.19	0.063
BMI	0.34	<0.001	0.29	<0.001	0.31	<0.001	0.27	<0.001	-0.16	0.023	0.14	0.054	-0.02	0.804
Systolic BP	0.34	<0.001	0.28	<0.001	0.24	0.001	0.14	0.045	-0.20	0.005	-0.10	0.178	-0.11	0.113
Diastolic BP	0.25	<0.001	0.23	0.001	0.24	0.001	0.16	0.022	-0.16	0.020	-0.04	0.604	-0.14	0.049
FPG	0.69	<0.001	0.57	<0.001	0.58	<0.001	0.41	<0.001	-0.37	<0.001	-0.21	0.003	-0.07	0.292
HbA1c	0.60	<0.001	0.51	<0.001	0.54	<0.001	0.30	<0.001	-0.39	<0.001	-0.23	0.001	-0.01	0.834
Total cholesterol	0.15	0.040	0.04	0.562	-0.10	0.167	0.00	0.967	-0.08	0.236	-0.19	0.007	0.06	0.378
Triglyceride	0.32	<0.001	0.27	<0.001	0.25	<0.001	0.18	0.011	-0.29	<0.001	-0.19	0.007	-0.06	0.361
HDL-C	-0.35	<0.001	-0.33	<0.001	-0.32	<0.001	-0.24	0.001	0.22	0.002	0.13	0.065	0.06	0.380
LDL-C	0.14	0.054	0.04	0.539	-0.09	0.208	0.01	0.871	-0.05	0.516	-0.16	0.023	0.07	0.339
Fasting insulin	0.30	<0.001	0.42	<0.001	0.37	<0.001	0.24	0.001	-0.33	<0.001	-0.18	0.010	-0.14	0.052
HOMA-IR	0.50	<0.001	0.52	<0.001	0.49	<0.001	0.34	<0.001	-0.36	<0.001	-0.21	0.003	-0.15	0.039
HOMA-B	-0.33	<0.001	-0.21	0.003	-0.23	0.001	-0.14	0.052	0.07	0.346	0.09	0.223	0.00	0.973

In the present study, we applied multivariate linear regression with the forward selection method to determine the independent predictors of these biomarkers. Entry criteria for the multivariate linear regression analysis included variables with p-value ≤ 0.10 in the univariate regression analysis (Table 2), and the results are presented in Table 3. CRP and IL-6 were independently predicted by FPG and HbA1c, while FPG and fasting insulin autonomously estimated TNF-α. FPG and BMI independently forecasted serum cortisol levels, whereas adiponectin’s autonomous predictors comprised HbA1c and fasting insulin. The independent predictors of IL-10 included HbA1c and TC, while IGF-1 was autonomously determined by HOMA-IR.

**Table 3:** Multivariate linear regression analysis of pro- and anti-inflammatory markers. Multivariate linear regression by the forward selection method was used with an entry criterion of P ≤ 0.10 and a removal criterion of P > 0.10 in univariate linear regression. CRP: C-reactive protein; FPG: fasting plasma glucose; HbA1c: glycated hemoglobin; TNF-α: tumor necrosis factor-alpha; TC: total cholesterol; IGF-1: insulin-like growth factor 1; HOMA-IR: homeostasis model assessment of insulin resistance; BMI: body mass index; IL-10: interleukin-10.

	β-value	t-value	P-value
CRP			
FPG	0.53	7.53	<0.001
HbA1c	0.22	3.18	0.002
TNF-α			
FPG	0.48	7.32	<0.001
Fasting insulin	0.19	2.88	0.004
IL-6			
FPG	0.39	4.82	<0.001
HbA1c	0.27	3.35	0.001
Cortisol			
FPG	0.35	5.13	<0.001
BMI	0.14	2.07	0.040
Adiponectin			
HbA1c	-0.30	-4.27	<0.001
Fasting insulin	-0.20	-2.84	0.005
IL-10			
HbA1c	-0.214	-3.13	0.002
TC	-0.169	-2.46	0.015
IGF-1			
HOMA-IR	-0.146	-2.080	0.039

Binary logistic regression analysis was conducted to determine the risk of developing T2DM (Table 4). The risk of T2DM occurrence is enhanced with increasing levels of CRP (OR: 2.69), TNF-α (OR: 1.20), IL-6 (OR: 1.83), and cortisol (OR: 1.01). In contrast, it declined with increasing concentration of adiponectin (OR: 0.81) and IL-10 (OR: 0.88) in comparison to the control group (P < 0.05).

**Table 4:** Odds ratios of pro- and anti-inflammatory markers for T2DM risk. CI: confidence interval; OR: odds ratio; T2DM: type 2 diabetes mellitus; CRP: C-reactive protein; IL-6: interleukin-6; IL-10: interleukin-10; IGF-1: insulin-like growth factor 1; TNF-α: tumor necrosis factor-alpha.

Parameters	OR (95% CI)	P-value
CRP (μg/ml)	2.69 (2-3.61)	<0.001
TNF-α (pg/ml)	1.2 (1.14-1.26)	<0.001
IL-6 (pg/ml)	1.83 (1.55-2.16)	<0.001
Cortisol (ng/ml)	1.01 (1.01-1.02)	<0.001
Adiponectin (mg/mL)	0.81 (0.75-0.88)	<0.001
IL-10 (pg/mL)	0.88 (0.8-0.95)	0.002
IGF-1 (ng/ml)	1 (0.99-1)	0.500

**Figure 1:**
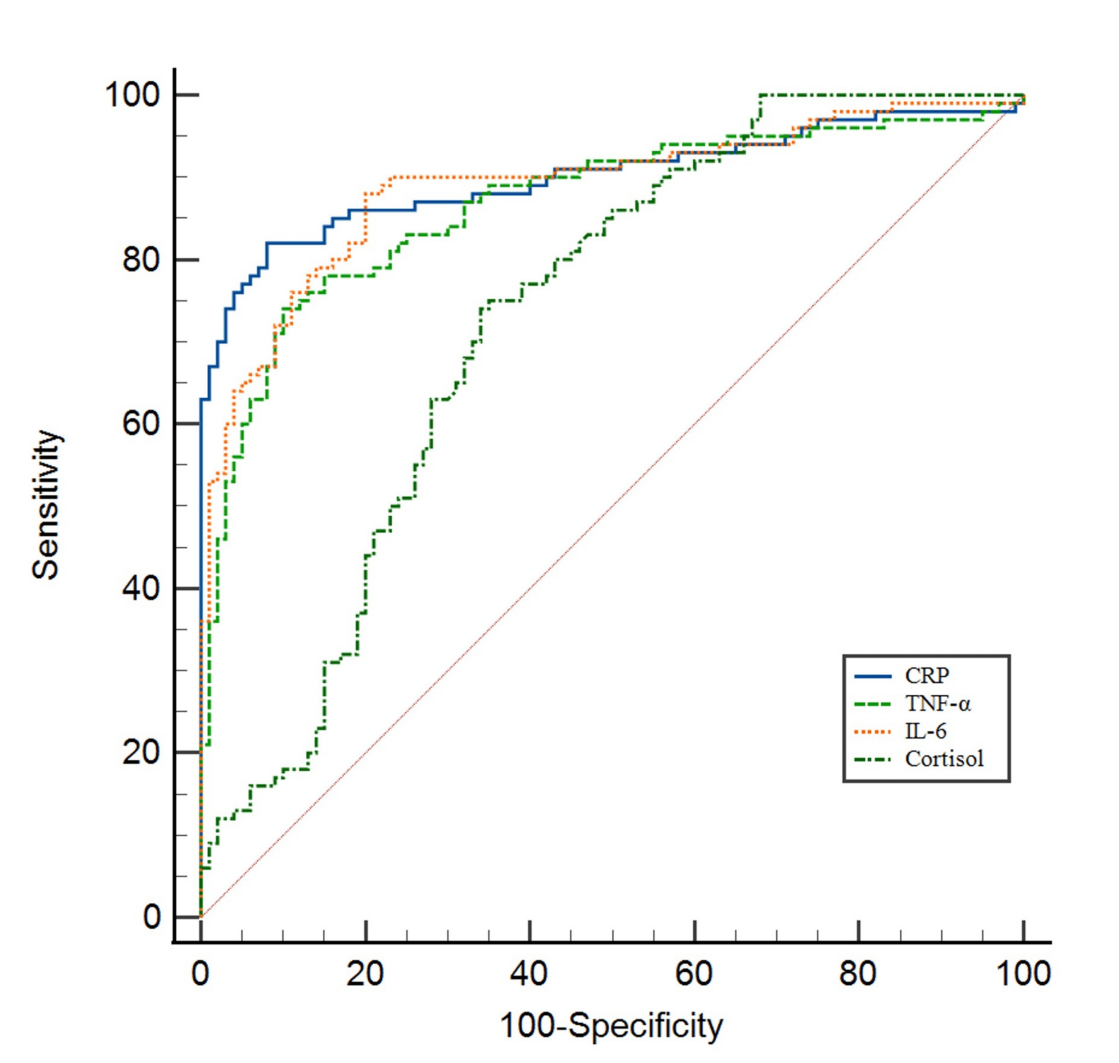
Receiver operating characteristics curve (ROC) illustrating the diagnostic utility of pro-inflammatory biomarkers in predicting type 2 diabetes mellitus (T2DM) in the geriatric population. CRP: C-reactive protein; TNF-α: tumor necrosis factor-alpha; IL-6: interleukin-6.

**Figure 2:**
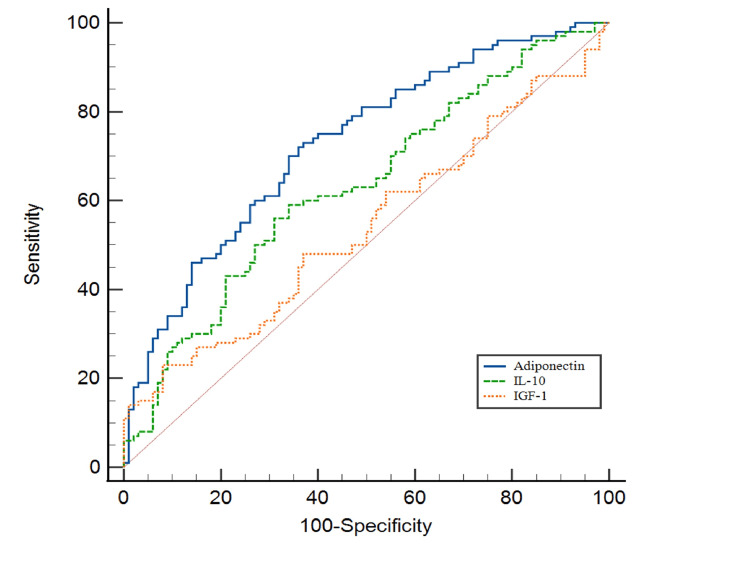
Receiver operating characteristics curve (ROC) illustrating the diagnostic utility of anti-inflammatory biomarkers in predicting type 2 diabetes mellitus (T2DM) in the geriatric population. IL-10: interleukin-10; IGF-1: insulin-like growth factor 1.

Receiver operating characteristic (ROC) curve analysis was performed to ascertain the diagnostic accuracy of these biomarkers in predicting T2DM (Table 5, Figures 1, 2). CRP, TNF-α, IL-6, cortisol, adiponectin, and IL-10 exhibited statistically significant area under curve values (P < 0.05), while IGF-1 did not (P=0.327). The optimal cutoff values of these biomarkers were determined by the Youden index. CRP (>3.80 µg/ml) exhibited 82% sensitivity and 92% specificity; TNF-α (>22.90 pg/ml) had 74% sensitivity and 90% specificity; IL-6 (>4.67 pg/ml) showed 88% sensitivity and 80% specificity; cortisol (>103.15 ng/ml) displayed 75% sensitivity and 65% specificity; adiponectin (≤7.42 µg/ml) demonstrated 70% sensitivity and 66% specificity; IL-10 (≤5.19 pg/ml) revealed 56% sensitivity and 69% specificity; and IGF-1 (≤107.24 ng/ml) had 23% sensitivity and 92% specificity. 

**Table 5:** Cut-off points and diagnostic utility of serum pro- and anti-inflammatory markers level for T2DM. AUC: area under curve; CI: confidence interval; T2DM: type 2 diabetes mellitus; CRP: C-reactive protein; TNF-α: tumor necrosis factor-alpha; IL-6: interleukin-6; IL-10: interleukin-10; IGF-1: insulin-like growth factor 1.

Parameters	Optimal cut-off value	AUC (95% CI)	P-value	Sensitivity (95% CI)	Specificity (95% CI)	Positive predictive value (PPV)	Negative predictive value (NPV)
CRP (μg/ml)	>3.80	0.90 (0.85-0.94)	<0.001	82 (73.1-89.0)	92 (84.8-96.5)	91.1 (84.0-95.2)	83.6 (77.0-88.6)
TNF-α (pg/ml)	>22.90	0.87 (0.81-0.91)	<0.001	74 (64.3-82.3)	90 (82.4-95.1)	88.1 (80.3-93.1)	77.6 (71.2-82.9)
IL-6 (pg/ml)	>4.67	0.89 (0.84-0.93)	<0.001	88 (80.0-93.6)	80 (70.8-87.3)	81.5 (74.7-86.8)	87.0 (79.5-92.0)
Cortisol (ng/ml)	>103.15	0.73 (0.66- 0.79)	<0.001	75 (65.3-83.1)	65 (54.8-74.3)	68.2 (61.6-74.1)	72.2 (64.3-79.0)
Adiponectin (mg/mL)	≤7.42	0.73 (0.66-0.79)	<0.001	70 (60.0-78.8)	66 (55.8-75.2)	67.3 (60.4-73.6)	68.7 (61.2-75.4)
IL-10 (pg/mL)	≤5.19	0.63 (0.56-0.70)	0.001	56 (45.7-65.9)	69 (59.0-77.9)	64.4 (56.2-71.7)	61.1 (54.8-67.0)
IGF-1 (ng/ml)	≤107.24	0.54 (0.47- 0.61)	0.327	23 (15.2-32.5)	92 (84.8-96.5)	74.2 (57.5-86.0)	54.4 (51.4-57.4)

## Discussion

This study aimed to investigate the diagnostic accuracy of pro- and anti-inflammatory biomarkers for T2DM and their correlation with clinical and biochemical parameters and to determine their independent predictors. The extensive analysis of these biomarkers showed that metabolic dysregulation, insulin resistance, and inflammation are intricately linked. The imbalance between pro- and anti-inflammatory pathways via the NF-κB pathway plays a central role in aging-associated inflammation and T2DM [1-3]. T2DM can be considered an accelerated model of aging, as T2DM and aging are linked at the molecular level by inflammation and insulin resistance, which leads to an increased risk of mortality [4,11].

In the present study, serum CRP, TNF-α, IL-6, and cortisol levels were elevated, while adiponectin and IL-10 were reduced in the T2DM group, and IGF-1 showed no significant difference. These findings are similar to de Rekeneire et al. (2006), who found elevated CRP, TNF-α, and IL-6 levels in T2DM [1]. The Jackson Heart Study reported elevated morning serum cortisol in T2DM, and another study by Radin et al. (2016) showed increased serum cortisol in elderly T2DM subjects [8,12]. Similar to our study, Coimbra et al. (2014) detected significantly lower adiponectin levels in the elderly T2DM group compared to the control group [5]. In the current study, serum IL-10 was significantly diminished in the T2DM group, and these findings align with a study conducted by Yaghini et al. (2011) in the Iranian population [6]. The Leiden 85-Plus Study showed a reduced IL-10 production capacity in the blood of T2DM subjects [13]. The NF-κB signaling pathway plays a central role in inflammation by regulating pro- and anti-inflammatory cytokines [14,15]. In the present research, serum IGF-1 level was lower in T2DM compared to the control group, but it did not reach a statistically significant level. These findings align with the study by Cortizo et al. (1998) and Aneke-Nash et al. (2016), who reported no significant difference in IGF-1 levels in T2DM and normoglycemic groups [9,16]. However, those with impaired fasting glucose (IFG) or combined IFG and impaired glucose tolerance showed significantly higher IGF-1 levels compared to normoglycemic subjects [16].

In the current study, CRP was positively correlated with BMI, blood pressure, glycemic parameters, lipid profile, serum insulin, and HOMA-IR. This finding aligns with a previous study conducted by Taniguchi et al. (2002) [17]. CRP was independently predicted by FPG and HbA1c, emphasizing that hyperglycemia plays a central and significant role in the inflammation associated with T2DM. This study showed that TNF-α and IL-6 were positively correlated with age, BMI, SBP, DBP, FPG, HbA1c, TG, fasting insulin, and HOMA-IR, and negatively correlated with HDL-C and HOMA-B. Our finding corresponds with a study by Ullah et al. (2021), who reported a positive association between TNF-α and BMI, HbA1c, and serum glucose, and a negative relationship with HDL-C [18]. Degirmenci et al. (2019) reported that elevated TNF-α and IL-6 levels are linked to both T2DM and insulin resistance [7]. In our study, FPG and fasting insulin independently predicted TNF-α levels, while FPG and HbA1c independently predicted IL-6 levels. This finding shows that elevated glucose and insulin levels upregulate the expression of pro-inflammatory markers, similar to the study conducted by Goyal et al. (2012) [19]. TLR4 signaling in adipocytes stimulates NF-κB, resulting in an increased pro-inflammatory gene expression (TNF-α, IL-1, IL-6, MCP-1) and decreased insulin sensitivity [20]. Our study revealed that serum cortisol was positively correlated with BMI, SBP, DBP, FPG, HbA1c, TG, fasting insulin, and HOMA-IR and was independently predicted by BMI and FPG. Ortiz et al. (2019) reported that an increase in serum cortisol was associated with elevated FPG and HbA1c in T2DM subjects, and elevated cortisol increased the odds of diabetes [12]. Cortisol, a hormone released in response to stress, regulates glucose and lipid metabolism and is linked to T2DM through insulin resistance and β-cell dysfunction. Cushing’s disease, a condition characterized by hypercortisolism, shows impaired insulin signaling, resulting in hyperglycemia and activate lipolysis, producing a condition similar to T2DM [21].

In the present study, adiponectin showed a significant positive correlation with HDL-C, while an inverse association with BMI, SBP, DBP, FPG, HbA1c, TG, fasting insulin, and HOMA-IR. The serum adiponectin levels were independently forecasted by HbA1c and fasting insulin. Mohammedsaeed et al. (2023) reported that adiponectin levels were inversely correlated with fasting blood glucose, BMI, and insulin resistance in metabolic syndrome [22]. Adiponectin improves tissue insulin sensitivity, and its decreased level is associated with insulin resistance and T2DM development. In this study, serum IL-10 levels were independently predicted by HbA1c and TC and negatively correlated with FPG, HbA1c, TC, TG, LDL-C, fasting insulin, and HOMA-IR. These findings align with those of Straczkowski et al. (2005), who reported a negative correlation between IL-10 and both fasting insulin and TG [23]. IL-10, an anti-inflammatory cytokine, counteracts the effects of TNF-α and IL-6 by enhancing insulin receptor tyrosine kinase signaling [24]. In the present study, IGF-1 was inversely correlated with DBP and HOMA-IR and independently predicted by HOMA-IR. The evolutionarily conserved insulin-like growth factor (IGF) axis plays a central role in aging and the pathogenesis of various aging-related diseases like osteoporosis and T2DM. This axis interacts with insulin signaling, affects glucose metabolism, and exacerbates insulin resistance [25,26].

In our study, binary logistic regression analysis identified the variable influences on T2DM susceptibility; higher levels of CRP, TNF-α, and IL-6 increased the odds of T2DM. Elevated adiponectin and IL-10 were protective against T2DM and reduced its probability. The findings of our study align with previous studies, indicating that elevated adiponectin and IL-10 lower the odds of T2DM [13,27]. Another study showed elevated CRP, TNF-α, and IL-6 in individuals aged 70-79 years increased the risk of T2DM. In another study, it was shown that subjects in the highest quartile of cortisol had a higher prevalence of T2DM compared to those in the lowest quartile [8,12]. These pro- and anti-inflammatory biomarkers highlight their potential importance as early indicators for T2DM.

This study's ROC curve analysis determined the diagnostic accuracy of pro-inflammatory and anti-inflammatory markers in T2DM. CRP, TNF-α, IL-6, cortisol, adiponectin, and IL-10 showed significant discriminatory effectiveness in diagnosing T2DM. These biomarkers' optimal cutoff values, sensitivity, and specificity can be utilized in clinical settings. A study in the Turkish population showed a significant AUC for CRP, with the optimal cutoff value for diagnosing T2DM based on FPG being 1.8 μg/ml (60% sensitivity and 57% specificity) for males and 2.5 μg/ml (60% sensitivity and 57% specificity) for females, respectively [28]. The optimal cutoff value in our study was >3.80 μg/ml with a sensitivity of 82% and specificity of 92%. Sinatora et al. (2022), in a study conducted on postmenopausal women diagnosed with metabolic syndrome according to IDF guidelines, found an AUC of 0.60, with a sensitivity of 46.1% and a specificity of 79.5% [29]. The study we performed, TNF-α demonstrated an AUC of 0.87, with a sensitivity of 74% and a specificity of 90% for diagnosing T2DM in the geriatric population. Abdella et al. (2018) reported an AUC of 0.74 with an optimal cutoff value of 7.5 μg/ml, with a sensitivity of 88% and specificity of 51% for diagnosing T2DM [30]. This aligns with our result, which showed an AUC of 0.73 for adiponectin with a cutoff value of ≤7.42 μg/mL, along with a 70% sensitivity and 66% specificity for diagnosing T2DM.

The present study has a few limitations; it is a case-control study with a limited sample size and conducted solely in the North Indian population. Moreover, we recommend interventional and longitudinal studies in diverse ethnic communities to validate our findings further and deepen the understanding of these biomarkers.

## Conclusions

In conclusion, our results reveal that elevated pro-inflammatory (CRP, TNF-α, IL-6, and cortisol) and decreased anti-inflammatory (adiponectin and IL-10) increased the odds of T2DM in the geriatric population. These biomarkers have diagnostic potential, promising advancements in clinical applications, and guidance for future therapeutic strategies. These biomarkers may facilitate the way of precision medicine in T2DM treatment by stratifying the subjects.
